# Tagus River microbial profile through nanopore sequencing on samples gathered from Prainha do Braco de Prata, Lisbon

**DOI:** 10.12688/openreseurope.18072.2

**Published:** 2024-12-05

**Authors:** Cristiano Pedroso-Roussado, Mariana Pestana, Ricardo Dias, Mónica Nunes, Pedro Pascoal, Marcelo Pereira, Nuno Nunes

**Affiliations:** 1 ITI/LARSyS, Universidade de Lisboa Instituto Superior Tecnico, Lisboa, Portugal; 2 BioISI-Biosystems and Integrative Sciences Institute, Universidade de Lisboa Faculdade de Ciencias, Lisbon, Portugal; 3 cE3c-Centre for Ecology, Evolution and Environmental Changes & CHANGE-Global Change and Sustainability Institute, Universidade de Lisboa Faculdade de Ciencias, Lisbon, Portugal

**Keywords:** Tagus River, Freshwater microbiota, Nanopore sequencing, Microbiota, Lisbon

## Abstract

**Background:**

Freshwater ecosystems play a vital role for hosting life, and their study can elucidate their dynamic state throughout time. However, there is not much knowledge about the microbial profiles and their relevance for the ecosystem balance is still unclear.

**Methods:**

In this Brief Report three freshwater samples collected in the Tagus River north margin were analysed through 16S-targeted nanopore sequencing and by customized bioinformatics pipeline.

**Results:**

Our results revealed a consensual microbial profile with Candidatus *Pelagibacter*, *Egibacter*, and *Ralstonia* as the most abundant genera. Additionally, through a literature review we found that the ecosystem services provided by these genera are mostly related to organic matter decomposition.

**Conclusions:**

Despite the need for a more robust sampling and analyses, we conclude that there is potential to use microbial profile approaches to help define the relevant microbial biomarkers to clarify the ecosystem services in the Tagus River freshwater ecosystem.

## Introduction

A complete comprehension of the Tagus River ecosystem is lacking. Due to the transboundary relevance of the Tagus River, its comprehension is paramount. Moreover, there is also insufficient information about the present and future impact posed by challenges in water management ( Sondermann & de Oliveira, 2022), by climate change ( Sondermann & de Oliveira, 2022), and anthropogenic alterations ( Fernandes *et al.*, 2020).

In terrestrial ecosystems most biomass comes from plants, where microbial biomass accounts for ~70% in the ocean ( Bar-On *et al.*, 2018). Microorganisms have short lifecycles and large population size so their organization and structure correspond to a swift adaptation to the shifting environmental changes ( Cavan *et al.*, 2019; Collins *et al.*, 2014; Hellweger *et al.*, 2014). The global climate crisis is pressing the changes in marine ecosystems threatening the services they provide. This happens by major variations in the species present in marine habitats. To assess such fast shifts unified frameworks need to be developed that uses data from ecology, metabolism, and climate change ( Baltar *et al.*, 2019). Without this knowledge, the ability to understand, evaluate, and forecast the future of marine communities, ecosystems, and their services is almost inefficient.

In this Brief Report we wanted to elucidate the microbial profile of a coastline location in the Tagus River estuary (Beato, Lisboa), and infer the potential causes and consequences of the presence of the observed taxa.

## Methods

### Samples collection

**Figure 1.**
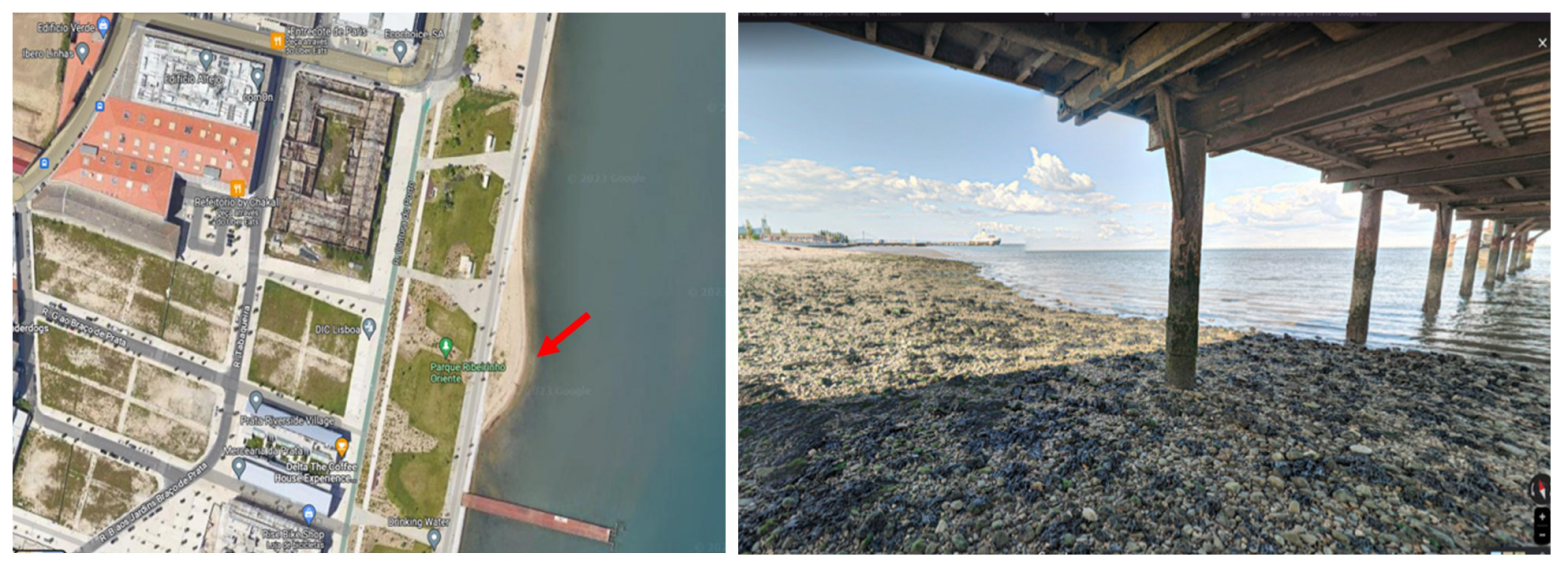
Sample collection site – ‘Praínha de Braço de Prata’. Left: aerial view map of the site (red arrow). Right: photo of the site taken in May 2020 by Miguel Silva. Google Maps (n.d.) (View from Praínha de Braço de Prata). Retrieved in July 13, 2023, from https://www.google.com/maps/place/38°44'45.9%22N+9°05'47.2%22W/.

The environmental water samples were collected in Praínha de Braço de Prata ( Figure 1). Three water samples were collected using three sterile plastic containers (5 L) for microbiota analysis. The containers were submerged at 0.5 meters and opened below surface to guarantee no air-borne microorganisms’ collection.

Total DNA was extracted from 5 L of each sample collected from the Tagus River and sequentially filtered through 20 μm, 3 μm, 0.45 μm, and 0.22 μm filters. The 0.45 μm and 0.22 μm filters were combined into a single 5 mL PowerWater Bead Pro Tube for DNA extraction using the DNeasy PowerWater Kit (Qiagen) according to the manufacturer's instructions. The purity of the extracted DNA was evaluated by measuring absorbance at 260 nm with a NanoDrop 1000 Spectrophotometer (Thermo Fisher Scientific, USA). The DNA was stored at −20 °C until further processing.

### Amplification of then 16S rDNA gene and sequencing library preparation

For the rDNA gene amplification, Long Amp hot start Taq 2× master mix (New England Biolabs, MA, USA) was used at 1X along with 50 ng/μL of genomic DNA from Tagus River water samples. To amplify the full-length 16S rDNA bacterial gene, 0.25 μM of the primer pair 27F (5′-AGAGTTTGATCMTGGCTCAG-3′) and 1492R (5′-CGGTTACCTTGTTACGACTT-3′) were used. Both PCRs were conducted on a Biometra UNO II, using the following conditions for 16S rDNA gene: 1 cycle of 94 °C for 1 min, 35 cycles of 94 °C for 20 s, 55 °C for 30 s, and 65 °C for 2 min, and a final extension of 65 °C for 5 min. Subsequently, amplification products were visualized via gel electrophoresis and purified using the Solid Phase Reversible Immobilization (SPRI) technique with magnetic beads (Stotchevoi *et al*., 2020). The library was prepared from 200fmol input DNA from each sample using the Sequencing Native Barcoding Kit 24 V14 (SQK-NBD114.24) (Oxford Nanopore Technologies, Oxford, UK) in accordance with the manufacturer’s protocol.

### Sequencing run

Sequencing runs were performed using R10.4.1 flow cells on a GridION sequencing platform and sequencing data were acquired in real time using MinKNOW 18.08.2 software. Sequencing data was stored in fastq files, with each file representing a batch of 4000 reads.

### Bioinformatics analysis

Sequencing data was derived from 16S amplicons, with the exclusion of low-quality reads. The remaining reads underwent size selection, retaining those with lengths between 1200 bps and 1700 bps, accomplished through *prinseq-lite* ( Schmieder & Edwards, 2011). Taxonomic classification employed a Lowest Common Ancestor approach, utilizing an index based on k-mers that map to the lowest common ancestor of all genomes known to encompass a specific k-mer.

The validation of our bioinformatics approach was performed using the ZymoBIOMICS™ Microbial Community Standard. Specifically, we sequenced this mock community using 16S rRNA amplicon sequencing and analyzed the data with the same pipeline applied to our samples. The proportions of sequencing reads classified to each taxon closely matched the expected proportions in the mock community. This agreement between the observed and expected proportions confirms the accuracy of our methodology in reflecting community composition, thereby validating the reliability of our approach for the samples studied.

For post-classification, the data underwent rarefaction and was subjected to several analyses: i) alpha-diversity group significance analysis ( Bolyen *et al*., 2019); and ii) sample dissimilarity analysis - Principal Coordinates Analysis (PCoA): Applied for beta-diversity analysis based on the Bray-Curtis similarity index. Taxa abundance was determined, employing a genera prevalence cutoff of ≥ 0.01 ( Bolyen *et al*., 2019). The sequencing depth for rarefaction was standardized to the sample with the lowest read count, which is 339,909 reads. This ensures that all samples are compared on an equal footing, avoiding biases introduced by differences in sequencing depth. In the rarefaction process reads were randomly subsampled to match the sequencing depth of the sample with the lowest read count (339,909 reads). This step was implemented to standardize the dataset while preserving the relative abundances of taxa within each sample.

## Results

### Nanopore sequencing general output

The three samples showed similar quality parameters which gives confidence that the sample collection and the sequencing approach were appropriately performed ( Table 1). Specifically, the mean read length is equal in the three analyses (1,570-1,574 bases), the same for the mean read quality (Q = 17.7-18.0), and the percentage of reads above the cutoffs. The sample 2 produced less reads (and bases) than the other two samples, but that did not hamper the quality of the output.

**Table 1.** Main nanopore sequencing quality parameters from the three environmental samples.

Quality feature (units)	Sample 1	Sample 2	Sample 3
Total number of reads (thousand)	>534	>339	>474
Total number of classified reads (%)	99.82	99.87	99.91
Total number of bases (million)	>841	>533	>746
Mean read length (bases)	1,574	1,570	1,571
Read length N50 (bases)	1,594	1,584	1,582
Mean read quality (Q score) *	17.5	18.0	18.0
Reads above quality 7 cutoff (%)	100.0	100.0	100.0
Reads above quality 10 cutoff (%)	100.0	100.0	100.0
Reads above quality 15 cutoff (%)	84.1	84.3	84.7

* Q score of 7 means a basecall accuracy of 80%, Q score of 10 means a basecall accuracy of 90%, and a Q score of 15 means an accuracy of 96.8%

### Prainha de Braço de Prata microbiota

The top main phylum, genus, and species observed in the three water samples are shown in Table 2.

**Table 2.** Three top phylum, genera, and species identified in the three samples with their respective relative abundance.

Sample	Rank	Taxa (relative abundance, %)
1	Phylum	*Pseudomonadota* (36), *Actinomycetota* (10), *Bacteroidota* (11)
	Family	*Flavobacteriaceae* (10), *Roseobacteraceae* (10), *Egibacteraceae* (7)
	Genus	*Egibacter* (7), *Tateyamaria* (6), *Ralstonia* (6)
	Species	*Egibacter rhizosphaerae* (7), *Tateyamaria omphalii* (6), *Ralstonia solanacearum* (6)
2	Phylum	*Pseudomonadota* (65), *Actinomycetota* (15), *Bacteroidota* (15)
	Family	*Burkholderiaceae* (14), *Roseobacteraceae* (14), *Flavobacteriaceae* (13)
	Genus	*Ralstonia* (14), Candidatus *Pelagibacter* (11), *Egibacter* (10)
	Species	*Ralstonia solanacearum* (14), Candidatus *Pelagibacter* sp. (11), *Egibacter rhizosphaerae* (10)
3	Phylum	*Pseudomonadota* (45), *Actinomycetota* (13), *Bacteroidota* (10)
	Family	*Pelagibacteraceae* (17), *Egibacteraceae* (10), *Flavobacteriaceae* (9)
	Genus	Candidatus *Pelagibacter* (17), *Egibacter* (10), *Ralstonia* (7)
	Species	Uncultivated bacterium from the SAR86 clade (16), *Ralstonia solanacearum* (10), *Egibacter* *rhizosphaerae* (7)

In order to assess the Praínha do Braço de Prata microbiota we qualified and quantified the shared taxa between the three samples ( Table 3).

**Table 3.** Shared taxa at the phylum, family, genus, and species level between the three samples. Only the taxa with relative abundances above 10% are shown.

Rank	TaxaID (Relative Frequency, %)
Phylum	*Pseudomonadota* (61) *Actinomycetota* (17) *Bacteroidota* (16)
Family	*Flavobacteriaceae* (16) *Pelagibacteraceae* (15) *Roseobacteraceae* (14) *Egibacteraceae* (13) *Burkholderiaceae* (12)
Genus	Candidatus *Pelagibacter* (15) *Egibacter* (14) *Ralstonia* (12)
Species	*Egibacter rhizosphaerae* (14) Candidatus *Pelagibacter* sp. FZCC0015 (14) *Ralstonia solanacearum* (12)

The microbial profiles differed between sample 1 and sample 3 at the phylum, genus, and species level (Shannon index; p-value < 0.05). The sample 1 and sample 2 showed similar alpha-diversities (Shannon index; p-value > 0.05). The samples showed incongruent dissimilarities microbial profiles amongst them. There were no differences amongst the three samples’ microbial profiles at the genus level. At the species level, the microbial profile from sample 3 is different from sample 1 and 2, but sample’s 2 and sample’s 1 microbial profile are similar. Lastly, he phylum level, the microbial profile from sample 2 is different from sample 1 and 3, and sample’s 1 shows a similar microbial profile than sample 3 (PERMANOVA R ^2^ = 1).

### Ecosystem services

Based on the shared taxa found between the three samples we used the top three genus and species to assess in PubMed database studies that inform the potential ecosystem services these taxa might be performing in the Tagus River. In total it was identified 17 articles related to *Pelagibacter* gen. and *Pelagibacter* sp., and 37 related to *Ralstonia* gen. and *Ralstonia solanacearum*. No article was found for *Egibacter* or *Egibacter rhizosphaerae*. Additionally, no review article was published about the ecosystem services of these taxa in the defined period of search in the systematic analysis. The relevant articles found follow as: 14 for *Pelagibacter* gen. and *Pelagibacter* sp., and 11 for *Ralstonia* gen. and *R. solanacearum* ( Table 4).

**Table 4.** Relevant ecosystem services present in literature for the most abundant taxa.

TaxaID	Relevant reference	Ecosystem service/ phenotype
Candidatus *Pelagibacter* or SAR11	Rivers *et al.,* 2013	Re-establishing the pre-oil spill microbial community structure.
	Tsementzi *et al.,* 2019	Oligotrophy; auxotrophy for reduced sulfur (lacking a sulfite oxidase); differential phage predation and/or adaptation (fine tuning) of core genes to environmental conditions (e.g. temperature).
	Liu *et al.,* 2023	Resistance to salinity; ability to grow in low nutrients sites (like dissolved inorganic nitrogen); utilizers of dissolved organic carbon.
	Han *et al.,* 2020	Heterotrophy; carbon cycling; generalista; dimethylsulfoniopropionate degradation.
	Cerro-Gálvez *et al.,* 2021	Oligotrophy; antitoxicity strategies (to anthropogenic dissolved organic carbon; high tolerance to hydrophobic chemicals
	Chun *et al.,* 2021	‘Skeleton’-formation and participate in species-engineered microenvironments; dominant in surface waters under stratified summer conditions; fine-tuning microbial modular structures; present in the nanoparticle-associated fraction; nitrate reduction, nitrification, dark sulfide oxidation.
	Delpech *et al.,* 2021	Oligotrophy; follow; abundant in low nutrient concentrations and in stratified surface waters phytoplankton blooms
	Sun *et al.,* 2021	Able to maximize nutrients uptake even when in low concentrations.
	Zhang *et al.,* 2022	Present in anticyclonic eddies, in warmer surface water temperatures, where there is less diverse bacterial communities; oligotrophy; survives at lower salinity and chlorophyl a concentrations but tolerate higher concentrations.
	Anderson & Harvey, 2022	Oligotrophy; rely on copiotrophs to assimilate sources of carbon and other metabolites.
	Fortin *et al.,* 2022	Predominant in *Alexandrium* sp. blooms; present in the particle-attached fraction of the surface water.
	Guo *et al.,* 2022	Involved in nitrogen and/ or sulfur cycling: prevalent in oxygen-rich surface oceans
	Larkin *et al.,* 2023	Heterotrophy; partition marine niche spaces; adapted to regions with low inorganic nitrogen supply; reveals habitat-specific partitioning in replication strategies for surface ocean communities
	Sun *et al.,* 2023	Prefers nitrogen-containing carbohydrates, oligo-glucans, oligo-cellulose, and lignin-derived aromatic fragments; polysaccharide degradation; scavenging mode in free-living fractions for the assimilation of dissolved carbohydrates, oligotrophy; key in significant flux of dissolved organic carbon and nutrient mineralization at the bottom of watery ecosystems.
*Ralstonia*	Coll *et al.,* 2020	Degradation of contaminants (e.g., organic pollutants);
	Rasool *et al.,* 2020	Tolerance to heavy metals like thallium; role in distributing metals; dominant in midstream soil samples; tent to enrich in bank of steam soils
	Chang *et al.,* 2021	Rare pathogenicity and transmitted through polluted waters.
	Liu *et al.,* 2023	Degrade microcystins; may enhance host-resistance to toxic cyanobacterial stress by affecting the whole bacterial community.
	Yang *et al.,* 2023a	May utilize diesel-derived organic intermediates produced by hydrocarbon degraders.
	Yang *et al.,* 2023b	Thrive in soil discharge and plant decomposition conditions; decomposition of several aromatic hydrocarbons.
*R. solanacearum*	Planas-Marquès *et al.,* 2020	Vascular pathogen; wide geographic distribution; colonizes many hosts, persists in soil and water for long periods; moves horizontally through the xylem ring by degrading the cell wall of primary xylem or pit membranes in the secondary xylem vessels.
	Biosca *et al.,* 2021	Infects ornamental plants; lytic lifestyle.
	Wen *et al.,* 2023	Cause wilt disease; affects the composition and diversity of rhizosphere microbiome

## Conclusion

Rivers present a key importance for human life and for nature balance since it is the primordial source of renewable water ( Vörösmarty *et al.*, 2010). However, there is a lack of studies that focus the microbial diversity in rivers compared to marine or lake ecosystems that could elucidate about its relevance for urban areas and ecosystems services. The dynamic flow of water and nutrients in rivers make their study complex but necessary to answer foundational questions regarding land use and novel threats like antimicrobial resistance and the emergence of invasive species ( Reddington *et al.*, 2020; van Rossum *et al.*, 2015). Despite the low number of samples analysed through this Brief Report, it is clear that we have insufficient information about the taxonomy of the most abundant taxa detected like *Pelagibacter* and *Egibacter* and more research is necessary to shed light on their relevant phenotypes aiming a better comprehension of the Tagus River ecosystem. Interestingly, the most abundant *Pelagibacter* strains were commonly found in marine waters rather than freshwaters (“FZCC0015” and “IMCC9063”). This observation constitutes a finding that deserves better attention since it may represent evidence of salinity changes in the Tagus River estuary.

## Ethics and consent

Ethical approval and consent were not required.

## Data Availability

BioProject Data Bank: 16S rRNA genomic data from the analyses of freshwater samples collected in Praínha do Braço de Prata (Lisbon, Portugal). Accession number: PRJNA1116853; https://www.ncbi.nlm.nih.gov/bioproject/?term=PRJNA1116853; available in the NCBI Sequence Read Archive ( Pedroso-Roussado *et al.*, 2024).
