# Supplementary material for: Tagus River microbial profile through nanopore sequencing on samples gathered from Prainha do Braco de Prata, Lisbon
Source: Open Res Eur. 2025 Apr 11;4:155. Originally published 2024 Jul 29. [Version 3] doi: 10.12688/openreseurope.18072.3 (PMC12015429; doi:10.12688/openreseurope.18072.3)
Supplement: Peer review history

# Reviewer report: Reviewer response for version 3

**DOI:** 10.21956/openreseurope.21786.r53067

**Published:** 2025-04-22

**Authors:** Sushanta Deb

**Affiliations:** 1 Washington State University, Pullman, WA, USA

The manuscript has been substantially improved from its previous version, with each of the concerns raised thoroughly addressed. It can now be accepted.

Is the study design appropriate and does the work have academic merit?

Yes

Is the work clearly and accurately presented and does it cite the current literature?

Yes

If applicable, is the statistical analysis and its interpretation appropriate?

Yes

Are all the source data underlying the results available to ensure full reproducibility?

Yes

Are the conclusions drawn adequately supported by the results?

Partly

Are sufficient details of methods and analysis provided to allow replication by others?

No

Reviewer Expertise:

Sequence analysis , metagenomics and comparative microbial genomics

I confirm that I have read this submission and believe that I have an appropriate level of expertise to confirm that it is of an acceptable scientific standard.

# Reviewer report: Reviewer response for version 2

**DOI:** 10.21956/openreseurope.20599.r48168

**Published:** 2025-01-24

**Authors:** Anyi Hu

**Affiliations:** 1 Institute of Urban Environment, Chinese Academy of Sciences, Xiamen, China

Thank author’s efforts for addressing my comments. One of my major concerns related to the occurrence of Pelagibacter was well explained in the revised version. However, I think it would be better to provide some environmental parameters, such as salinity etc., which can show the investigated area was under the influence of sea intrusion. By the way, the information of the introduction and conclusion sections, which highlighted the ecosystems service of ocean and river, are different. Based on the findings, I suggest may focus on the role of estuary and estuarine microbiome for ecosystem service.

Is the study design appropriate and does the work have academic merit?

Partly

Is the work clearly and accurately presented and does it cite the current literature?

Partly

If applicable, is the statistical analysis and its interpretation appropriate?

Not applicable

Are all the source data underlying the results available to ensure full reproducibility?

Partly

Are the conclusions drawn adequately supported by the results?

Partly

Are sufficient details of methods and analysis provided to allow replication by others?

Yes

Reviewer Expertise:

Aquatic microbial ecology

I confirm that I have read this submission and believe that I have an appropriate level of expertise to confirm that it is of an acceptable scientific standard, however I have significant reservations, as outlined above.

# Reviewer report: Reviewer response for version 2

**DOI:** 10.21956/openreseurope.20599.r49898

**Published:** 2025-01-23

**Authors:** Sushanta Deb

**Affiliations:** 1 Washington State University, Pullman, WA, USA

Manuscript titled "Tagus River microbial profile through nanopore sequencing on samples gathered from Prainha do Braco de Prata, Lisbon" provide valuable information about microbial composition of freshwater ecosystem. However manuscript need attention to following points before indexing.

 please mention how low quality reads were excluded from the raw sequence dataset.

 Provide a detailed explanation of the bioinformatics method, including how clean reads were mapped to the database and which database was used for taxonomic assignment.

 Replacing Table 3 with a bar chart depicting the microbial composition of the three samples would provide a more comprehensive and visually engaging presentation for the reader.

 Please discuss the taxons identified at higher levels in these three samples in the context of published studies on the microbial community composition of freshwater rivers.

Is the study design appropriate and does the work have academic merit?

Yes

Is the work clearly and accurately presented and does it cite the current literature?

Yes

If applicable, is the statistical analysis and its interpretation appropriate?

Yes

Are all the source data underlying the results available to ensure full reproducibility?

Yes

Are the conclusions drawn adequately supported by the results?

Partly

Are sufficient details of methods and analysis provided to allow replication by others?

No

Reviewer Expertise:

Sequence analysis , metagenomics and comparative microbial genomics

I confirm that I have read this submission and believe that I have an appropriate level of expertise to confirm that it is of an acceptable scientific standard, however I have significant reservations, as outlined above.

# Reviewer report: Reviewer response for version 1

**DOI:** 10.21956/openreseurope.19531.r42633

**Published:** 2024-08-19

**Authors:** Anyi Hu

**Affiliations:** 1 Institute of Urban Environment, Chinese Academy of Sciences, Xiamen, China

This report employed the third-generation (nanopore) sequencing to profile the taxonomic composition of bacterial communities in three surface water samples of the Tagus River. Candidatus *Pelagibacter*, *Egibacter*, and *Ralstonia* were identified as the main bacterial taxa, which were proposed to play an important role in maintaining the river ecosystem service (e.g., organic matter decomposition).

 This work is a preliminary descriptive study due to the limited sample size and shallow analysis. However, this report provided a snapshot for the taxonomic information of the planktonic bacterial communities in the Tagus River. Maybe in the future, more samples, more environmental variables and more advanced technologies (e.g., metagenomics) can be included into the Tagus River microbiome study.

 I have a concern on the discussion about the ecosystem service of *Pelagibacter. *Since *Pelagibacter *is a typical marine bacteria, please carefully confirm whether the riverine bacteria is a marine one or the freshwater taxa who has a closed relationship with *Pelagibacter. *In addition, I think the current dataset and analysis of this report is hard to infer the ecosystem service provided by planktonic bacteria.

Is the study design appropriate and does the work have academic merit?

Partly

Is the work clearly and accurately presented and does it cite the current literature?

Partly

If applicable, is the statistical analysis and its interpretation appropriate?

Not applicable

Are all the source data underlying the results available to ensure full reproducibility?

Partly

Are the conclusions drawn adequately supported by the results?

Partly

Are sufficient details of methods and analysis provided to allow replication by others?

Yes

Reviewer Expertise:

Aquatic microbial ecology

I confirm that I have read this submission and believe that I have an appropriate level of expertise to confirm that it is of an acceptable scientific standard, however I have significant reservations, as outlined above.

# Reviewer report: Reviewer response for version 1

**DOI:** 10.21956/openreseurope.19531.r42629

**Published:** 2024-08-13

**Authors:** Dave R. Clark

**Affiliations:** 1 University of Essex, Colchester,, UK

The study by Pedroso-Roussado and colleagues characterises the microbial community structure of three water samples from the estuarine part of the Tagus River, Portugal. Although this is a low number of samples for a typical microbial ecology study, the authors have not over-interpreted their data nor conducted any under-powered statistical analysis. The authors report on the most abundant taxa detected, and infer their roles in organic matter degradation, highlighting the need for further data to better understand the role of understudied taxa. The study appears interesting, but some further basic information is required, particularly in the methods and results to provide further context.

**Abstract:**

 Generally fine, but a few examples of odd / unclear phrases that may need revision. For instance, it is not clear what the authors mean by “Freshwater ecosystems play a vital role for humans and more-than humans”. If the authors mean that freshwater ecosystems are globally important, then it is better to say this in simple terms. It is also not clear what the authors mean by ‘consensual microbial profile’ – some re-wording is needed here.

**Introduction:**

 Again, generally good, although it might have been nice to have a little more background on the Tagus River and why it is worthy of further study, as the introduction is a little too focussed on purely marine science rather than estuarine environments.

**Methods:**

 There are some missing details on the molecular methods here. For example, how was DNA extracted from the samples? Were 16S genes amplified by PCR, if so, which primers?

 Some additional details in the bioinformatics are also needed: “The used approach had been validated through ZymoBIOMICSTM Microbial Community Standard” – how was this approach validated and what data do the authors have to show this approach works? Also, to what sequencing depth were the samples rarefied to? Also, what steps are involved in this approach? At the moment, this methodology is not reproducible.

**Results:**

 Again, there is some basic but interesting information about the samples missing. What was their diversity? How similar (beta diversity) were the samples to each other?

 Also, whilst the literature search on ecosystem function was useful, why did the authors not use a bioinformatic tool to do this (e.g. PiCRUST2 or similar)?

Is the study design appropriate and does the work have academic merit?

Yes

Is the work clearly and accurately presented and does it cite the current literature?

Yes

If applicable, is the statistical analysis and its interpretation appropriate?

Not applicable

Are all the source data underlying the results available to ensure full reproducibility?

Partly

Are the conclusions drawn adequately supported by the results?

Yes

Are sufficient details of methods and analysis provided to allow replication by others?

Partly

Reviewer Expertise:

Microbial community ecology, bioinformatics, statistical ecology

I confirm that I have read this submission and believe that I have an appropriate level of expertise to confirm that it is of an acceptable scientific standard, however I have significant reservations, as outlined above.

# Response

**Published:** 2024-11-25

**Authors:** Cristiano Pedroso-Roussado

**Affiliations:** Universidade de Lisboa Instituto Superior Tecnico, Lisbon, Lisbon, Portugal

The authors want to thank Dr Dave Clark and Dr Anyi Hu for their kind revisions of your Brief Report. 

 We took into consideration all their comments and proceed to address them in full throughout this new version.

 In summary, we remade the methods' section and now we believe it fully shows how the experimental work was conducted. 

 We greatly appreciated the comment of Dr Hu about the Pelagibacter marine or freshwater provenance. We checked in the dataset and indeed the two strains are described as marine bacteria, while here they were detected in estuarine Tagus River samples. This observation raises questions about the ecosystem dynamics of the Tagus River, and it deserves further exploration.

 Finally, we hope that our response is of reviewers' and editors' appreciation and that our Brief Report can now be approved.

 Thank you again
